# Cardioembolic Stroke: Past Advancements, Current Challenges, and Future Directions

**DOI:** 10.3390/ijms25115777

**Published:** 2024-05-26

**Authors:** Yuji Kato, Kenta Tsutsui, Shintaro Nakano, Takeshi Hayashi, Satoshi Suda

**Affiliations:** 1 Department of Neurology and Cerebrovascular Medicine, Saitama Medical University International Medical Center, Hidaka 350-1298, Japan; thayashi@saitama-med.ac.jp (T.H.); sudasa@saitama-med.ac.jp (S.S.); 2 Department of Cardiology, Saitama Medical University International Medical Center, Hidaka 350-1298, Japan; tsutsuik@saitama-med.ac.jp (K.T.); snakano@saitama-med.ac.jp (S.N.); 3 Department of Cardiology, Teikyo University School of Medicine, Tokyo 173-8605, Japan

**Keywords:** atrial fibrillation, embolic stroke of unknown source, direct oral anticoagulants, atrial cardiopathy

## Abstract

Cardioembolic stroke accounts for over 20% of ischemic strokes and is associated with worse outcomes than other types of strokes. Atrial fibrillation (AF) is the most common risk factor for cardioembolic stroke. In this narrative review, we present an update about cardioembolic stroke mainly related to AF and atrial cardiopathy. Direct oral anticoagulants (DOACs) have revolutionized stroke prevention in patients with AF; however, their efficacy in preventing recurrent embolic stroke of unknown source remains uncertain. Various cardiac monitoring methods are used to detect AF, which is crucial for preventing stroke recurrence. DOACs are preferred over warfarin for AF-related stroke prevention; however, the timing of initiation after acute ischemic stroke is debated. Resuming anticoagulation after intracerebral hemorrhage in AF patients requires careful assessment of the risks. While catheter ablation may reduce the incidence of cardiovascular events, its effect on stroke prevention is unclear, especially in heart failure patients. Atrial cardiopathy is the emerging cause of embolic stroke of unknown source, which indicates atrial structural and functional disorders that can precede AF. Future research should focus on refining stroke risk prediction models, optimizing AF detection, understanding the roles of ablation and anticoagulation in stroke prevention, and establishing atrial cardiopathy as a therapeutic target, which could significantly reduce the burden of stroke.

## 1. Introduction

Cardioembolism is the cause of at least 20% of all ischemic strokes [1]. Cardioembolic strokes are associated with worse neurological deficits and worse outcomes than other types of strokes [2]. While atrial fibrillation (AF) is the most common source of cardioembolism, interventional cardiology procedures and cardiac disorders including ventricular akinesia, valvular heart disease, acute myocardial infarction, endocarditis, and monogenic cardiovascular diseases may also be responsible. The introduction of direct oral anticoagulants (DOACs) has significantly transformed stroke prevention in patients with AF; however, a more comprehensive understanding of long-term DOAC treatment is needed.

Most cryptogenic strokes, or strokes of unknown cause, are thought to be thromboembolic in origin; accordingly, the concept of embolic strokes of unknown source (ESUS) was introduced to describe strokes in which the cause was undetermined despite an adequate diagnostic workup [1]. However, DOACs were not more effective than aspirin in preventing recurrent stroke in patients with ESUS in two randomized controlled trials [3,4], which has prompted a re-evaluation of the ESUS concept and demonstrates the importance of an aggressive approach to obtain a definitive diagnosis of stroke etiology.

The Atrial Cardiopathy and Antithrombotic Drugs in Prevention After Cryptogenic Stroke (ARCADIA) trial showed that the risk of recurrent stroke did not significantly differ between patients with cryptogenic stroke and atrial cardiopathy treated with the DOAC apixaban and those treated with aspirin [5]. Initial hypotheses suggested potential benefits, especially in high-risk subgroups like patients with atrial cardiopathy, associated with an elevated risk of stroke. However, the findings of the ARCADIA trial temper these expectations. Much remains to be understood about the connections between AF, atrial cardiopathy, and the risk of stroke—further research is warranted. This narrative review aims to summarize the features of cardioembolic stroke and discuss the detection of AF and optimal management of patients with cardioembolic stroke mainly related to atrial fibrillation and atrial cardiopathy.

## 2. Heart Disease and Stroke

Ischemic stroke is frequently associated with underlying heart disease. Although systemic hypoperfusion related to heart failure or during heart surgery is responsible in some cases [6], the main cause of cardiogenic stroke is cardioembolism, which accounts for 20% or more of all ischemic strokes [1]. When all heart diseases are included, more strokes can be attributed to heart disease (described later in this chapter).

(1) Features of cardioembolic stroke

More than 50% of cardioembolic strokes are sudden in onset. In contrast, atherothrombotic and lacunar strokes have a sudden onset in 30% and 20% of cases, respectively [7]. Lack of stepwise progression is also a feature of cardioembolic stroke [8]. Because embolism tends to affect large or cortical arteries, aphasia and hemispatial neglect are more common in cardiogenic stroke than in other subtypes [7]. Furthermore, disturbance of consciousness is more prominent, and stroke severity as measured by the National Institutes of Health Stroke Scale (NIHSS) is significantly worse with cardioembolic stroke than with atherothrombotic and lacunar strokes [7]. The neurological deficit with embolic stroke is typically greatest just after onset, and some cases show rapid recovery, or “spectacular shrinking deficit”, which reflects embolus migration. In one study of major hemispheric stroke, 13 of 14 patients with spectacular shrinking deficit had a cardiogenic embolism [9].

Radiological features of cardioembolic stroke include massive infarction and multiple infarctions which affect both hemispheres or the anterior and posterior circulation territories combined. Large artery occlusion and hemorrhagic transformation are common in cardioembolic stroke [10]. In a study that examined factors associated with lack of early recanalization after intravenous thrombolysis in patients with an occluded cerebral artery, the prevalence of AF was lower among those who experienced early recanalization [11]. The clinical and radiological features of cardioembolic stroke are summarized in Table 1.

(2) Heart diseases which cause embolic stroke

**Figure 1:**
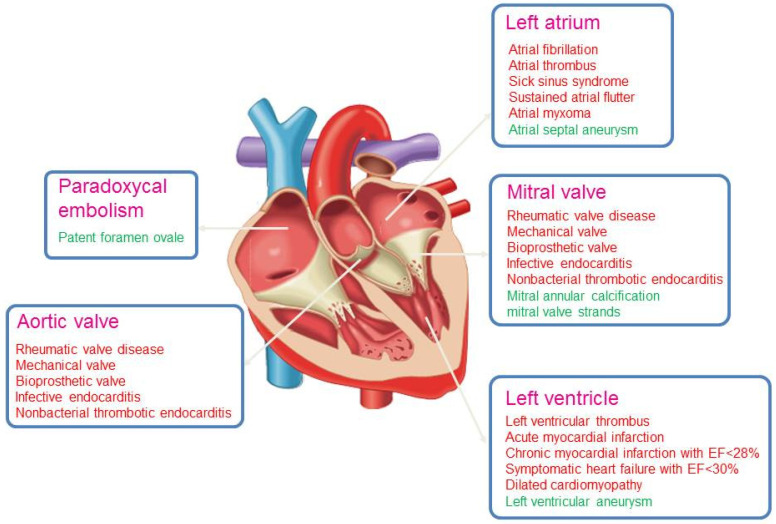
Heart diseases associated with stroke. Diseases associated with a high risk of stroke are shown in red text; green text indicates low or uncertain risk. EF, ejection fraction.

Some heart diseases have a strong association with embolic stroke while others have only a weak or even questionable association. Furthermore, approximately 50% to 70% of stroke patients have at least one possible cardioembolic source [12]. Even when heart disease is present, it may be difficult to determine whether it is truly the cause of stroke or only an incidental comorbidity. Because the classification of stroke type using the Trial of Org 10172 in Acute Stroke Treatment (TOAST) system has suboptimal inter-rater reliability, the Stop Stroke Study (SSS)-TOAST algorithm was designed in 2005, which showed excellent accuracy and inter-rater reliability (kappa statistic, 0.90) [13]. In this algorithm, the relative risk of stroke is taken into account, which is classified as high, low, or uncertain (Figure 1). High risk was separated from a low-risk mechanism using an arbitrary 2% annual or one-time primary stroke risk threshold [13]. The clinical and radiological features of stroke subtypes are incorporated as well. A web-based version [14] can be used online (https://ccs.mgh.harvard.edu/ccs_title.php, accessed on 1 May 2024). The agreement between the web-based version and the original TOAST system is moderate (kappa statistic, 0.59) [15]. AF is the most frequent cause of cardioembolic stroke [16] and is discussed in detail below.

(3) Cryptogenic stroke

Up to 25% of ischemic strokes are cryptogenic [1]. Embolism is generally considered the major cause. Thrombi removed from patients with cryptogenic stroke contain a high proportion of fibrin, as do thrombi from those with cardioembolic stroke [17]. Many stroke patients have at least one heart condition that has at least a small potential to cause strokes [12]. Furthermore, AF is eventually detected in many stroke patients who do not exhibit it at the time of admission [18]. Clinicians should be aggressive in seeking out heart disorders in patients with cryptogenic stroke. However, simply detecting AF does not necessarily mean that it was the cause [19]. This issue will be discussed in detail below.

## 3. Detection of AF and Patient Management

Detection of AF in a stroke patient, which is often transient and asymptomatic, is challenging yet crucial for managing and preventing AF-related cardioembolism.

(1) Mode of AF detection

Although 12-lead electrocardiography (ECG) is accurate, the recording time is brief; therefore, it has high specificity but low sensitivity for the detection of AF. Implantable devices like loop recorders and pacemakers exhibit high sensitivity and specificity but are invasive and not widely available. Holter monitors, which can track heart activity for 24 h to 2 weeks, provide a middle ground.

(2) Relationship between duration of monitoring and AF detection

The implantable loop recorder (ILR) has a significantly higher AF detection rate than non-invasive ECG and is able to conduct ECG monitoring for 2 to 3 years. It is particularly useful in asymptomatic patients. The effectiveness of extended cardiac monitoring in detecting AF in post-stroke patients has been emphasized in recent studies. The Cryptogenic Stroke and Underlying Atrial Fibrillation study reported that ILR detected new AF in 8.9% of patients within 6 months and in 12.4% within 12, far exceeding the 1.4% to 2.0% detection rates associated with standard ECG monitoring [18]. The 30-Day Cardiac Event Monitor Belt for Recording Atrial Fibrillation after a Cerebral Ischemic Event trial found that AF detection rates in patients with cryptogenic stroke who underwent 30-day and 24 h monitoring were 16.1% and 3.2%, respectively (*p* < 0.001) [20]. Similarly, the Effect of Implantable vs. Prolonged External Electrocardiographic Monitoring on Atrial Fibrillation Detection in Patients with Ischemic Stroke trial showed that the AF detection rate was significantly higher with 12-month implantable ECG monitoring than with 30-day external monitoring [21]. Finally, the Holter-Electrocardiogram-Monitoring in Patients with Acute Ischaemic Stroke trial reported AF detection rates of 14% and 5% for repeated 10-day Holter monitoring and traditional 24 h Holter monitoring (*p* = 0.002) [22]. These studies clearly demonstrate the greater effectiveness of prolonged and implantable monitoring methods for identifying AF. However, further research is needed to determine if extended cardiac monitoring enhances long-term outcomes in these patients.

(3) Patient management is individualized and based on the risk of stroke and the presence of AF

Patients who experience stroke have a high risk of recurrence. Therefore, it is critical to select appropriate antithrombic treatment (aspirin or a DOAC) in these patients, particularly when AF has not been detected. Recent guidelines suggest that the use of an ILR is reasonable to detect AF in those who have no known history of AF [23].

In contrast, in patients with a relatively low risk of stroke (e.g., low CHA2DS2-VASc score), screening may be conducted with a sequential step-up strategy. This strategy has demonstrated an increase in the detection rate of new AF in patients with ischemic cerebral infarction: 7.7% on admission, 5.1% during hospitalization, 10.7% at the first outpatient visit, 16.9% at the second with ECG or ILR [24].

**Figure 2:**
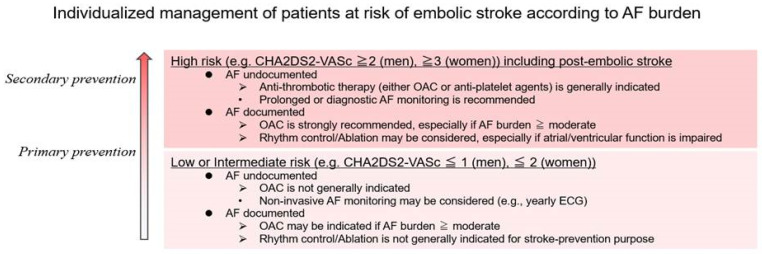
Patient management is based on risk of stroke and presence of atrial fibrillation.

Studies have consistently shown that cardiac monitoring of longer duration and higher frequency increases AF detection; however, such monitoring also captures short AF episodes which may be clinically insignificant. This raises the following question: do these episodes warrant anticoagulation? In general, anticoagulation should be reserved for patients who would benefit, considering the potential adverse effects. Our patient management recommendations are shown in Figure 2. The type of cardiac monitoring in stroke patients with no evidence of AF is based on stroke risk. Treatment decision making is based on stroke risk, presence of AF, and duration of AF episodes. This is particularly relevant as emerging technology enhances detection capability, leading to a surge of AF that would have been missed if modern detection modalities such as ILR had not been available. Given that the left atrial appendage is the most common site for intracardiac thrombi formation, surgical or percutaneous removal of the left atrial appendage is a feasible treatment option for patients at high risk of bleeding who require prevention of thromboembolism. With the development of innovative devices, transcatheter closure of the left atrial appendage has become increasingly prevalent in these situations [25].

The validity of extrapolation of these findings based on CHA2DS2-VaSc remains unclear in some regions that use the CHADS2 score system, and future studies must fill the gap.

Antithrombotic treatment and the intensity or invasiveness of atrial fibrillation (AF) detection depend on the intrinsic thrombotic risk and any history of prior embolic stroke. For patients who have experienced an embolic stroke, secondary prevention includes anticoagulation and, if feasible, ablation if AF has been documented. If AF remains undetected, aggressive screening should continue to optimize the therapeutic strategy. Primary prevention of stroke in patients with AF is tailored based on the burden of AF and the patient’s risk of stroke.

## 4. Anticoagulation

(1) Oral anticoagulation for preventing stroke in patients with non-valvular AF (NVAF)

Since its introduction in the 1950s, warfarin has been the cornerstone of thromboembolism prevention in patients with deep vein thrombosis, AF, or a prosthetic valve. Although warfarin is inexpensive, the therapeutic window is narrow and frequent monitoring is required. Moreover, it has interactions with many different foods and other drugs, and patient adherence to treatment can be poor. Oral anticoagulation therapy considerably changed in the 2010s with the introduction of DOACs for preventing stroke in patients with NVAF and for the treatment of venous thromboembolism. DOACs include direct thrombin inhibitors and inhibitors of factor Xa. Results from RCTs have shown that all four DOACs (dabigatran, rivaroxaban, apixaban, and edoxaban) are at least as effective as warfarin in preventing stroke and systemic embolism, and are approximately 50% less likely to cause symptomatic intracerebral hemorrhage (ICH) [26,27,28,29]. Although the use of DOACs without routine monitoring and frequent dose adjustment has been shown to be safe and effective, there may be clinical circumstances in specific patients when measurement of the anticoagulant effects of DOACs is required. Liquid chromatography–tandem mass spectrometry should be considered the gold standard test for measuring DOAC concentration [30], but it is difficult to implement in a clinical setting. The monitoring indices for dabigatran, a thrombin inhibitor, are the dilute TT (dTT) and the Ecarin clotting time (ECT). For Xa inhibitors, the anti-FXa assay correlates very well with plasma concentrations. Recent guidelines recommend DOACs as a first-line drug for the prevention of embolism in patients with NVAF [23,31,32].

(2) Early DOAC initiation after acute ischemic stroke in patients with AF

DOACs reduce the risk of ischemic stroke and systemic embolism in patients with AF. However, whether the timing of initiation influences the risks of recurrent thromboembolism and ICH after acute ischemic stroke is unclear. Early DOAC initiation may increase the risk of ICH; later initiation may increase the risk of stroke recurrence. Warfarin is associated with a paradoxical increase in the risk of ischemic stroke in the first 7 days of use, probably because of a transient hypercoagulable state caused by deactivation of protein C and protein S [33]. Given the lack of high-quality evidence, guideline recommendations regarding the timing of anticoagulation initiation after ischemic stroke have varied. The current recommendation in several guidelines is to delay initiation. The European guidelines suggest that the timing of anticoagulation initiation should be based on stroke severity as assessed by the NIHSS score: patients with minor, moderate, and severe stroke should receive anticoagulation on days 3, 6, and 12, respectively, after stroke onset [34]. However, a recent study in Japan that combined data from two prospective multicenter observational studies of patients with NVAF and stroke or transient ischemic attack found that initiating DOACs within 1 day after transient ischemic attack, 2 days after mild stroke, 3 days after moderate stroke, and 4 days after severe stroke decreased the risk of stroke without causing an increase in the incidence of major bleeding; the authors also reported similar findings in an external validation analysis of patients from six European registries [35]. The Timing of Oral Anticoagulant Therapy in Acute Ischemic Stroke With Atrial Fibrillation randomized trial showed that early DOAC initiation (within 4 days of stroke onset) was noninferior to delayed initiation (5–10 days) [36]. The Early versus Later Initiation of Direct Oral Anticoagulants in Post-Ischemic Stroke Patients with Atrial Fibrillation randomized trial studied DOAC initiation within 48 h of stroke onset in patients with minor or moderate stroke and on day 6 or 7 in those with major stroke and used imaging-based criteria to define stroke severity; one of the findings was that the incidence of symptomatic ICH was low [37]. However, the median NIHSS scores in the two trials noted above were relatively low (4 and 5, respectively). Forthcoming data from two ongoing randomized trials (NCT02961348 and NCT03021928) should add to the current evidence and will help guide clinical practice in the future.

(3) Resumption of anticoagulation after ICH in patients with AF

Balancing the risks of thromboembolism and bleeding is the primary concern when making decisions regarding anticoagulation therapy in patients with AF who have experienced ICH. Unfortunately, there is little evidence to help guide clinicians who encounter this conundrum, as shown by the wide variation in practice patterns. A 2014 study of AF patients who experienced ICH in five institutions in Europe reported that the proportion of patients who resumed antithrombotic drugs differed between institutions by as much as four-fold [38]. Current knowledge is largely based on retrospective observational studies. Because all randomized controlled trials investigating the benefit of anticoagulation in patients with AF excluded patients with a history of ICH [26,27,28,29], there is no consensus regarding the safety and timing of anticoagulation resumption in patients with prior ICH. The American Heart Association/American Stroke Association guidelines suggest considering resumption 7 to 8 weeks after spontaneous ICH in patients with NVAF to prevent thromboembolic events after weighing the benefits and risks (class IIB recommendation). The Japanese guidelines are in agreement but do not specify the timing. The European Heart Rhythm Association Practical Guide recommends restarting anticoagulation in selected patients with AF and ICH at least 4 weeks after the ICH event [31]; however, the quality of evidence supporting this recommendation is poor and it is uncertain whether it has translated into clinical practice. Prospective observational data from a Japanese study in which anticoagulants were resumed in 68% of patients and the median time to resumption was 7 days after ICH suggest that early resumption in patients with NVAF after ICH onset is safe [39]. When anticoagulation was restarted, warfarin was switched to a DOAC, and DOACs were switched to another DOAC type. Further large-scale studies with long-term follow-up are needed to determine the indications for resuming DOACs in AF patients with ICH as well as the optimal timing of resumption and the optimal type of agent. A recent meta-analysis of 412 patients with AF and ICH from four randomized trials reported that initiating oral anticoagulation reduced the risk of stroke or cardiovascular death [40]. Ongoing randomized and observational clinical trials will hopefully provide more data in the future (Table 2).

## 5. Catheter Ablation

(1) Maintaining sinus rhythm is the key to health

Achieving and keeping sinus rhythm in patients with AF, either through medication or ablation, is linked to a lower risk of stroke and other cardiovascular events. Treating AF when it is still paroxysmal may reduce the risk of stroke, as shown in the Fushimi AF registry study [41]. Maintaining sinus rhythm in patients with AF is associated with a significant reduction in the incidence of a composite of death from cardiovascular causes, stroke, or hospitalization for heart failure or acute coronary syndrome, and 81% of the treatment effect of early rhythm control is explained by sinus rhythm at 1 year after intervention [42,43]. These findings confirm that rhythm control should be achieved as early as possible. However, the use of antiarrhythmic drugs to maintain sinus rhythm is associated with higher rates of mortality and serious adverse events [44,45]. As a result, pulmonary vein isolation ablation has become the standard intervention for rhythm control [46]. Catheter ablation improves symptoms; reduces the incidence of major adverse cardiac events, mortality, and cardiovascular hospitalizations; and exhibits positive effects on left ventricular ejection fraction, left atrial size, and related biomarkers [47,48,49,50].

(2) Ablation effect on stroke incidence

However, ablation has not been shown to significantly reduce the incidence of stroke in randomized controlled trials. Possible explanations for this include underpowered studies and/or a low incidence of stroke in the study populations. In the Effect of Catheter Ablation vs. Antiarrhythmic Drug Therapy on Mortality, Stroke, Bleeding, and Cardiac Arrest Among Patients With Atrial Fibrillation trial, the incidence of the primary composite endpoint of death, disabling stroke, serious bleeding, or cardiac arrest was similar between ablation (8%) and medical therapy (9.2%); however, ablation was associated with a lower rate of death or cardiovascular hospitalization (51.7% vs. 58.1%; hazard ratio [HR] 0.83, 95% confidence interval [CI], 0.74–0.93; *p* = 0.002) [47]. A meta-analysis of data pooled from this trial and other large registries showed that the composite risk of death, stroke, and hospitalization for heart failure was significantly lower with ablation than with medical therapy alone [51]. A study of AF patients from the Swedish National Patient Register indicated that all-cause mortality (HR 0.51; 95% CI, 0.41–0.63) and incidence of stroke (HR 0.75; 95% CI, 0.53–1.07) were lower in patients who underwent ablation than in those who did not; however, the findings were not significant [52]. In a meta-analysis of 78,966 AF patients, the pooled risk of a cerebrovascular event was significantly lower in patients who underwent ablation than in those who did not (2.3% vs. 5.5%; risk ratio 0.57; 95% CI, 0.46–0.70; *p* < 0.001; I^2^ = 62%) [53].

Although catheter ablation in patients with AF appears to prevent stroke, further critical appraisal is necessary to objectively judge the cost-effectiveness balance of the “ablation as stroke prevention” concept, especially given the underwhelming number of patients needed to treat (NNT) (Table 3) [47,51,52,53].

(3) Relationship between heart failure and incidence of stroke

Because heart failure is considered a risk factor for stroke in patients with normal sinus rhythm and those with AF [54,55,56], and ablation of AF improves outcomes and biomarkers in patients with heart failure, it is tempting to speculate that ablation would be particularly useful in the heart failure population. However, the degree of stroke reduction that ablation provides in heart failure patients has not been determined. Further studies are needed to examine this important question.

## 6. Atrial Cardiopathy

**Figure 3:**
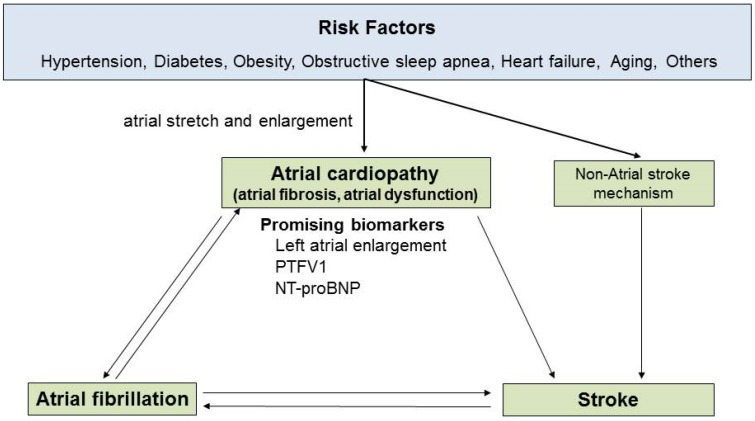
Atrial cardiopathy as a stroke risk factor. The causes of thromboembolic stroke include atrial fibrillation as well as atrial cardiopathy, which is caused by underlying changes in the atrial tissue that occur as a result of stretching and enlargement. Atrial cardiopathy in conjunction with an irregular heart rhythm exacerbates the tissue changes and impairs left atrial contraction, which increases the risk of thromboembolic stroke. Once stroke occurs, autonomic and inflammatory changes in the atrium may transiently increase the risk of atrial fibrillation.

Multiple stroke risk factors such as hypertension, diabetes, obesity, and obstructive sleep apnea contribute to left atrial stretching and enlargement [57,58]. Over time, this leads to fibrosis, scarring, and dysfunction of the left atrium [57,58]. The presence of these atrial abnormalities in the absence of AF is called ‘atrial cardiopathy’, a term used to describe atrial structural and functional disorders that can precede AF [59]. Atrial cardiopathy may serve as a precursor to AF or even be an independent factor associated with the development of atrial thrombus and subsequent stroke [59,60,61] (Figure 3). Diagnostic criteria for atrial cardiopathy have not been fully established, but it is usually diagnosed clinically using several biomarkers. Markers of atrial disease, such as left atrial enlargement on echocardiography, increased P-wave terminal force in lead V1 on ECG, and increased serum concentration of N-terminal pro-brain natriuretic peptide, are associated with increased stroke risk and appear to be promising biomarkers for atrial cardiopathy [59,62].

Atrial cardiopathy is considered one of the mechanisms of ESUS. Two previous trials in patients with ESUSs [3,4] did not account for the presence of atrial cardiopathy and failed to show that DOACs are superior to aspirin for preventing recurrent strokes. In contrast, the ARCADIA trial was restricted to ESUS patients with atrial cardiopathy, which shed light on a biologically distinct stroke mechanism with specific therapeutic implications [63].

In the ARCADIA trial, atrial cardiopathy was defined as P-wave terminal force > 5000 µV × ms in lead V1, serum N-terminal pro-brain natriuretic peptide > 250 pg/mL, or left atrial diameter index ≥ 3 cm/m^2^ on echocardiography [63]. Patients were randomized to apixaban twice daily or aspirin once daily. The diagnosis of atrial cardiopathy was based on N-terminal pro-brain natriuretic peptide in 61.1% of patients, P-wave terminal force in lead V1 in 53.4%, and left atrial diameter index in 1.3% [5]. The incidence and hazard rates of recurrent stroke of any type did not significantly differ between the apixaban and aspirin groups [5]. Similarly, the incidence rates of recurrent ischemic stroke/systemic embolism and recurrent stroke/all-cause death did not differ between the groups as well. The lack of differences between the apixaban and aspirin groups might be due to the fact that various disorders were included in atrial cardiopathy. The current criteria for atrial cardiopathy do not fully reflect the conditions that would benefit from anticoagulation. More suitable patient selection and thresholding of biomarkers that suggest atrial cardiopathy which would benefit from anticoagulation need to be clarified.

## 7. Future Directions

Future research should continue assessing the relationship between heart diseases and stroke while ascertaining AF. Furthermore, various biomarkers of atrial cardiopathy should be compared and perhaps combined to optimize the prediction of stroke risk. Ultimately, establishing the validity of atrial cardiopathy as a therapeutic target will require randomized clinical trials. Given that stroke accounts for 11.6% of deaths worldwide and imposes a substantial burden of disability [64], future studies of subclinical AF or atrial cardiopathy as a modifiable stroke risk factor may have a substantial impact on public health.

## Figures and Tables

**Table 1:** Features of cardioembolic stroke.

Clinical	Sudden Onset
	Lack of stepwise progression
	“Spectacular shrinking deficit”
	Disturbance of consciousness
	Cortical signs (aphasia, hemispatial neglect)
	High NIHSS score
Radiological	Massive infarction
	Multiple infarctions in different arterial territories
	Large artery occlusion

NIHSS, National Institutes of Health Stroke Scale.

**Table 2:** Ongoing clinical trials on resumption of antithrombotic therapy or LAAC after ICH.

Study Name	Country	Study Design	Intervention	Primary Outcome	Delay between ICH and Randomization, Days
SAFE-ICH	Japan	Multicenter, prospective, observational	No	Composite of these incidence rates up to 30 days after ICH: - Symptomatic ICH - Symptomatic stroke - All-cause death	1–14
A3ICH	France	Multicenter, phase 3, randomized, open-label, masked outcome assessment (PROBE), single (Outcomes Assessor)	Apixaban vs. LAAC vs. no anticoagulant or LAAC	Composite of all fatal or nonfatal major cardiovascular/cerebrovascular ischemic or hemorrhagic intracranial/extracranial events	30
ASPIRE	United States	Multicenter, phase 3, randomized, double-blinded, parallel assignment, quadruple (Participant, Care Provider, Investigator, Outcomes Assessor)	Apixaban compared with aspirin	Incidence of stroke of any type (ischemic or hemorrhagic) or death from any cause	15–180
ENRICH-AF	Canada	Multicenter, phase 3, randomized, open, blinded end point (PROBE), single (Outcomes Assessor)	Edoxaban compared with no anticoagulant	Stroke of any type and major hemorrhage as defined by ISTH criteria	15
PRESTIGE-AF	Europe	Multicenter, phase 3b, randomized, parallel-group, open, blinded end point (PROBE), single (Outcomes Assessor)	DOAC (all types) compared with no anticoagulant	Time to the first ischemic stroke event and time to first recurrent ICH event	15–180

DOAC, direct oral anticoagulant; LAAC, left atrial appendage closure; ICH, intracerebral hemorrhage; ISTH, International Society on Thrombosis and Haemostasis.

**Table 3:** Possibility of stroke reduction by AF ablation.

Study	Patients	Intervention	Control	Outcome
CABANA—Packer (2019) (Ref. [47])	n = 2204, symptomatic patients with AF aged 65 years and older or younger than 65 years with 1 or more risk factors for stroke	Ablation	Medical therapy (both rhythm and rate control)	Possible stroke reduction (statistically nonsignificant)
Barra (2018) (Ref. [53])	Meta-analysis of 13 studies including CABANA, n = 25,129 for ablation, 53,837 for medical treatment	Ablation (n = 25,129)	Medical therapy (n = 53,837)	Lower risk of cerebrovascular events (2.3% vs. 5.5%; RR = 0.57, 95%CI 0.46–0.70, *p* < 0.001)
				NNT 31
Akerstrom (2024) (Ref. [52])	Swedish National Patient Registry n = 48,786	Ablation	Medical therapy, propensity-matched	Possible stroke reduction (HR 0.75, 95% CI 0.53 to 1.07).
Saglietto (2020) (Ref. [51])	Meta-analysis of CABANA and 8 matched registries	Ablation (n = 241,372)	Medical therapy (n = 213,661)	Reduction in stroke (HR, 0.63; 95% CI, 0.56–0.70; I^2^ = 23%; NNT = 59) in 3.5 years
